# Advances in Nitric Oxide Signalling and Metabolism in Plants

**DOI:** 10.3390/ijms24076397

**Published:** 2023-03-29

**Authors:** Weibiao Liao, Abir U. Igamberdiev, José M. Palma

**Affiliations:** 1 College of Horticulture, Gansu Agricultural University, 1 Yinmen Village, Anning District, Lanzhou 730070, China; 2 Department of Biology, Memorial University of Newfoundland, St. John’s, NL A1C 5S7, Canada; igamberdiev@mun.ca; 3 Department of Stress, Development and Signaling in Plants, Spanish National Research Council, CSIC, 18008 Granada, Spain; josemanuel.palma@eez.csic.es

More than 15,000 scientific articles published since the late 1950s related to RNS action or detection in various plant materials are listed in the Web of Science database [1]. Nitric oxide (NO), as a major gaseous signaling molecule, may be involved in a series of plant physiological processes, ranging from seed germination to root development to seedling growth, and participate in plant response to abiotic stress, including drought stress, low-temperature stress, heat stress, high-salinity stress, ozone stress, and heavy metal stress [2]. In plants, NO is generated through two pathways: enzymatic and non-enzymatic reaction pathways [3]. In general, endogenous NO is produced mainly through oxidation and reduction pathways [4]. In the oxidation pathway, nitric oxide synthase (NOS)-like enzyme catalyzes the NO production from L-arginine, and nitrites are converted via the side reaction of nitrate reductase (NR) in the reduction pathway for NO production. Additionally, abiotic conditions also stimulate the release of endogenous NO. Apart from NR and NOS, the production of NO is also catalyzed by other enzymes. Of course, we shall not elaborate much further here.

Concerning the role of NO in postharvest fruit senescence, many important questions remain to be further studied and answered. For instance, the specific molecular mechanisms activated during NO-regulated fruit ripening and the signal transduction pathways by which NO interacts with other signaling molecules at the transcriptional or translational level remain unclear. Suitable concentrations of NO do alleviate the senescence in postharvest fruit, attributed to eight main factors based on the investigations by Li et al., including (1) ethylene biosynthesis, (2) the antioxidant system, (3) polyamine metabolism and γ-aminobutyric acid (GABA) shunt, (4) cell wall metabolism, (5) sugar metabolism, (6) energy metabolism, (7) the CRT/DRE-binding factor (CBF) pathway, and (8) *S*-nitrosylation [5]. Zhu et al. also summarized that an exogenous NO donor is involved in delaying postharvest senescence in horticultural crops by regulating a set of metabolisms, including ethylene biosynthesis, respiratory metabolism, cell wall metabolism, reactive oxygen species(ROS) metabolism, and energy metabolism [6]. Recently, Qian et al. discovered that NO inhibited quality deterioration and extended the postharvest life of water bamboo shoots by maintaining the integrity of mitochondrial ultrastructure and improving mitochondrial energy metabolism [7]. Additionally, the processes occurring during RNS (NO)-induced alleviation of seed dormancy have been investigated in depth. Moreover, reactive oxygen metabolism can act as a dormancy-breaking agent in seed germination [1].

In recent years, research hotspots on NO are still mainly focused on the response to abiotic stresses. NO might reduce cadmium (Cd) toxicity in plants and enhance plant resistance to Cd stress. The NO accumulation induced by Cd stress could also aggravate Cd toxicity in plants. However, the literature on the relationship between Cd stress and NO is controversial. The main reason for these conflicting results may be that NO production has multiple sources and multiple functional properties [8]. Certainly, the complex network of regulatory mechanisms of NO in response to Cd stress still needs to be further investigated. Wei et al. indicated that NO, as a ubiquitous gas signaling molecule, enhanced photosynthetic capacity and regulated endogenous hormonal equilibrium to alleviate salt toxicity in tomato seedlings [9]. The overexpression of phytoglobin *GmPgb1* in soybean attenuated the accumulation of foliar Na^+^ and limited ROS-induced damage in salt-stressed leaf tissue [10]. Chammakhi et al. found that NO was accumulated in the nodules of faba bean (*Vicia faba* L.) under drought and salt stresses [11]. However, as summarized by Shang et al., studies should integrate the existing fragmented pathways to establish a primary NO signaling pathway for salt resistance [12]. Then, the molecular mechanism of NO response to plant salt tolerance was further explored, such as the identification of *S*-nitrosylated target proteins or the effect of de-nitrosylation and transnitrosylation on plant salt tolerance. Zafari et al. demonstrated that under normoxia, *NtAOX* overexpression resulted in decreased nitric oxide (NO) levels; while under hypoxia, *AOX* overexpressors exhibited higher NO and *S*-nitrosylation levels than knockdowns [13]. Furthermore, transcriptome sequencing technology is also an important method to explore in terms of plant NO biology. Transcriptome data showed that increased peptidase inhibitor gene expression was the main reason for reduced tomato yellow leaf curl virus content and improved tomato resistance in NO treatment [14]. NO signaling plays a dual role in plant photosynthesis and photoprotection. High levels of NO stimulated the degradation of LONG HYPOCOTYL 5 (HY5) protein in tomato plants and further inactivated the transcription of genes encoding protochlorophyllide oxidoreductase C (PORC) and phytoene synthase 2 (PSY2)—two enzymes that catalyze the rate-limiting steps in chlorophyll and carotenoid biosynthesis. This study provides new insight into the mechanism of NO signaling in modulating HY5-mediated photosynthetic pigment biosynthesis at the transcriptional level [15]. In addition, NO is a key player in the signal transduction process of dark-induced Arabidopsis stomatal closure. COP1 transduced H_2_O_2_ signaling and promoted NO accumulation in guard cells by suppressing *FT*, *TSF* and *SOC1* expression levels, thus consequently leading to stomatal closure in darkness [16]. These findings add new insights into the mechanisms of dark-induced stomatal closure.

Much research work has been aimed at elucidating the most obvious physiological phenomena in NO response to plant growth and development and adversity stress, and what we lack most now is the discovery of a comprehensive regulatory network at the molecular level. It is clear that much remains to be discovered to obtain a clear regulatory network of NO signaling and metabolism in plants.
